# Imaging Features of Lung Ground-Glass Nodules and Their Correlation With Biological Behavior

**DOI:** 10.7759/cureus.97775

**Published:** 2025-11-25

**Authors:** Weikuan Xue, Bin Pan, Fuhui Huang, Fubin Zhang, Gang Li, Jinchun Chen

**Affiliations:** 1 Radiology, Rui'an People's Hospital, Ruian, CHN; 2 Internal Medicine, Rui'an People's Hospital, Ruian, CHN

**Keywords:** biological behavior, ground-glass nodules, imaging features, molecular markers, pathological types, prognosis assessment

## Abstract

Ground-glass nodules (GGNs) in the lungs are common and clinically significant manifestations in pulmonary imaging, drawing significant attention because of their potential for malignant transformation. With the popularization of low-dose CT screening, the detection rate of GGNs has significantly increased; however, their imaging characteristics are complex and diverse, posing challenges for clinical diagnosis and management. Current research mainly focuses on the imaging features of GGNs, such as size, shape, edge characteristics, and dynamic changes, gradually revealing their intrinsic connections with pathological types and molecular biological markers. However, there are still controversies and gaps in research regarding the specific correlations between imaging features, tumor biological behavior, and prognosis. This paper conducts a literature search using the PubMed/MEDLINE database with keywords including GGO, alveolar wall shadows, part-solid nodules, and subsolid nodules to find relevant studies. The terms used include GGO, GGN, and alveolar wall nodules. It analyzes the latest research progress on the imaging characteristics of lung GGNs and their biological behavior, systematically exploring their application value in pathological typing, molecular marker expression, and clinical prognosis assessment. The aim is to provide scientific evidence for clinical risk stratification, individualized treatment, and follow-up strategy formulation, thereby promoting the early diagnosis and precise treatment of lung cancer.

## Introduction and background

Ground-glass nodules (GGNs), as important imaging manifestations of early-stage lung cancer, have seen a significant increase in detection rates in recent years with the widespread adoption of low-dose CT (LDCT) screening. GGNs typically appear as ground-glass opacity on lung CT images, encompassing both pure GGNs (pGGNs) and mixed GGNs (mGGNs) with partial solid components [1,2]. Based on imaging features, GGNs are closely associated with various pathological types of lung adenocarcinoma, exhibiting diverse biological behaviors ranging from benign inflammation and non-invasive adenocarcinomas such as adenocarcinoma in situ (AIS) and minimally invasive adenocarcinoma (MIA) to invasive adenocarcinoma (IAC) [3,4]. Therefore, accurately distinguishing the benign or malignant nature of GGNs and their degree of invasiveness is crucial for guiding clinical management and personalized treatment.

With the promotion of LDCT screening, the detection rate of pulmonary GGNs has risen markedly, particularly among Asian female non-smokers, and patients with a family history of lung cancer show significantly increased risks of persistent, multiple, and growing GGNs [5]. The imaging characteristics of GGNs, including nodule size, density, proportion of solid components, margin morphology, lobulation, spiculation, and vascular signs, are all correlated with their pathological types and biological behaviors [6,7]. For instance, larger nodule size, higher CT values (e.g., >-410 Hounsfield units (HU)), higher proportion of solid components, presence of vacuole signs, and pleural retraction signs are more indicative of IAC [3,4]. Additionally, detailed analysis of vascular abnormalities and the relationship between nodules and pulmonary vessels on CT imaging can further improve the accuracy of malignancy assessment for GGNs [8,9]. In recent years, multiparameter imaging feature extraction based on deep learning and radiomics has been employed to construct predictive models, enhancing the non-invasive diagnostic capability for distinguishing benign and malignant GGNs [10,11]. These models, combined with clinical parameters and whole-lung imaging features, have achieved area under the curve (AUC) values exceeding 0.88, demonstrating promising clinical potential [10].

The biological diversity of GGNs is reflected not only in their pathological subtypes but also in their underlying mechanisms of malignant transformation. Single-cell transcriptomic studies reveal significant differences in tumor microenvironment (TME) composition and cellular functional states between GGNs and solid nodules, with GGNs exhibiting lower cellular proliferation activity and higher immune cell activation, suggesting a relatively lower risk of malignant progression [12,13]. Histological and molecular biological studies further identify specific gene expression patterns and driver gene mutations in GGNs, such as CCNE1 gene variants associated with malignant transformation [14], while functional remodeling of the immune microenvironment is also considered a key mechanism in the progression of GGNs to invasive lung adenocarcinoma [15,16]. These biological investigations provide a molecular basis for understanding the diversity of GGNs and their mechanisms of malignant transformation.

Research on the correlation between imaging features and biological behaviors is essential for achieving precise diagnosis and personalized treatment. By integrating multimodal approaches such as radiomics, artificial intelligence (AI)-assisted diagnosis, and liquid biopsy, the accuracy of predicting malignant risk in GGNs has improved, while effectively avoiding overdiagnosis and overtreatment [17-19]. Moreover, advancements in LDCT technology and image reconstruction algorithms, such as deep learning reconstruction (DLR) and photon-counting detector CT (PCD-CT), have significantly enhanced the accuracy of GGN volume measurement and reduced radiation doses, facilitating long-term follow-up and dynamic evaluation [20,21]. Concurrently, AI-powered automated lung nodule detection and classification systems have demonstrated high sensitivity and specificity in multicenter clinical data, further advancing the intelligent clinical management of GGNs [22,23].

In summary, as key imaging manifestations of early-stage lung cancer, GGNs have seen a marked rise in detection rates with the widespread use of LDCT screening. Detailed analysis of GGN imaging features, combined with in-depth research on biological behaviors, helps elucidate their diversity and potential mechanisms of malignant transformation. By exploring the intrinsic links between imaging features and biological behaviors, supplemented by advanced radiomics, AI, and molecular biomarker detection, precise diagnosis and personalized treatment of GGNs can be achieved, providing scientific evidence for clinical practice and optimizing patient outcomes. Moving forward, systematic review of relevant research progress, coupled with the latest data, will deepen the understanding of the imaging-biological correlation in GGNs and drive the refinement of precision screening and treatment strategies for early-stage lung cancer [7,10,17,24].

## Review

Imaging characteristics analysis of pulmonary GGNs

**Figure 1:**
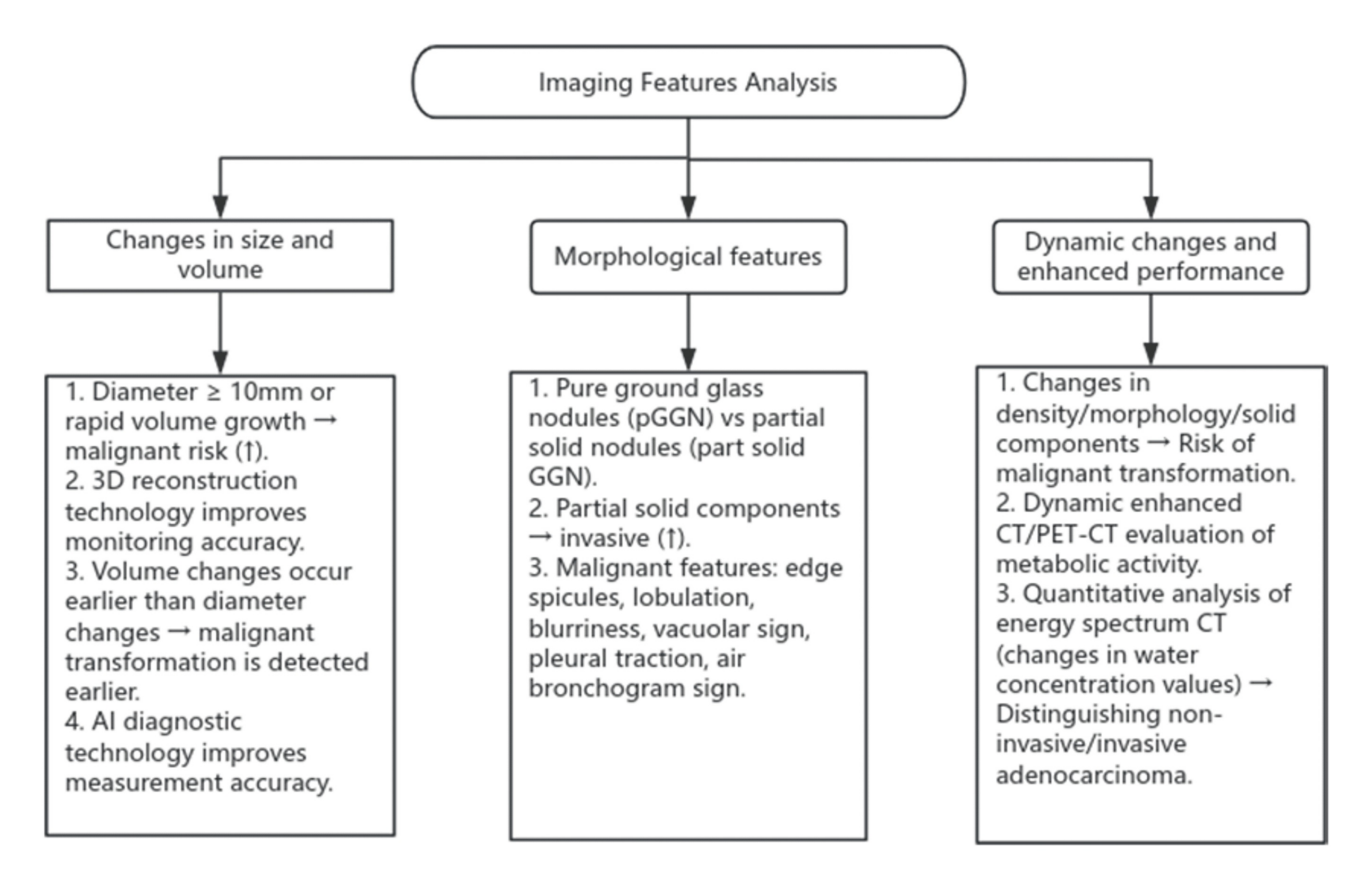
Imaging Features Analysis The flowchart displays the imaging features of ground-glass nodules in the lungs, created by Weikuan Xue (2025) from Rui'an AI: artificial intelligence; 3D: three-dimensional

The imaging characteristics analysis of pulmonary GGNs is presented in Figure 1.

Size and volume changes

The diameter and volume of pulmonary GGNs are critical indicators for evaluating their growth rate and malignant potential. Multiple studies have shown that when the nodule diameter exceeds 10 mm or the volume increases rapidly, the risk of malignancy significantly rises. For instance, in pGGNs, patients with nodules ≥ 10 mm in diameter have a higher incidence of IAC [25]. Moreover, volume measurements are more accurate than diameter measurements in reflecting nodule changes, especially when utilizing three-dimensional (3D) reconstruction techniques and semi-automated or automated segmentation methods, which enhance the accuracy of dynamic monitoring [26,27]. Different measurement methods yield varying size assessments, with Martin's mean diameter demonstrating higher inter-observer stability [28]. In dynamic monitoring, volume changes often precede diameter changes, enabling the earlier detection of potential malignant transformation [29]. Additionally, LDCT screening reveals that pGGNs grow slowly, making them suitable for long-term follow-up, whereas part-solid GGNs exhibit faster growth in both volume and diameter, warranting consideration for surgical intervention [30,31]. With the application of AI in pulmonary nodule diagnosis, faster and more precise assessments of nodule size and volume changes can provide accurate diagnostic results. Therefore, combining dynamic changes in nodule size and volume, particularly with advanced 3D reconstruction and automated measurement technologies, can more effectively predict the malignant risk of GGNs and guide clinical decision-making.

Morphological features

The morphological manifestations of GGNs are primarily classified into pGGNs and part-solid GGNs. The presence of solid components is closely associated with increased invasiveness and serves as an important imaging marker for malignancy [30,32]. Studies indicate that the five-year survival rate of part-solid GGNs is significantly lower than that of pGGNs, suggesting a poorer prognosis [30]. Furthermore, nodule margin characteristics are crucial for assessing malignant risk. Spiculated, lobulated, or indistinct margins are all suggestive of an increased likelihood of malignancy [4,33]. Additional diagnostic features on imaging include vacuole signs, pleural retraction, and air bronchograms [4]. Single-cell RNA sequencing studies reveal that cancer cells in GGNs exhibit downregulated proliferation signals but enhanced immune cell activation, indicating unique biological behavior [12]. Thus, morphological features combined with detailed imaging findings are vital for evaluating the biological behavior of GGNs and hold significant value in guiding clinical treatment strategies. While most literature analyzes the morphological features of pGGNs and part-solid GGNs, there is limited research on the correlation between features such as lobulation, spiculation, vacuole signs, pleural retraction, air bronchograms, and pathological outcomes.

Dynamic changes and enhancement patterns

The dynamic changes of GGNs are key to assessing their biological behavior. During follow-up, alterations in nodule density, morphology, and solid components can indicate the risk of malignant transformation [30,34]. Dynamic contrast-enhanced CT and PET-CT serve as auxiliary tools to evaluate metabolic activity and vascular supply, aiding in malignancy assessment [9]. A study using spectral CT for quantitative analysis of pGGNs found that changes in water concentration values during arterial and venous phases have high diagnostic value for distinguishing non-invasive from IAC [9]. Additionally, increases in solid components, worsening margin morphology, and rapid volume growth on imaging are closely linked to pathological progression [19,33]. Prolonged follow-up reveals significant increases in the proportion and density of solid components, suggesting heightened malignant risk [30]. Therefore, integrating dynamic imaging changes and enhancement patterns facilitates early identification of malignant transformation, enabling the formulation of appropriate intervention strategies to improve patient outcomes.

Biological behavior characteristics of pulmonary GGNs

**Figure 2:**
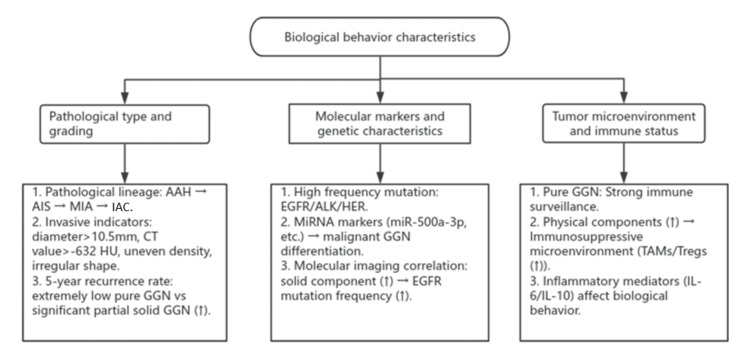
Biological Behavior Characteristics Flowchart showing the biological behavior of ground-glass nodules (GGNs) in the lungs, created by Weikuan Xue in Rui'an (2025) AAH: atypical adenomatous hyperplasia; AIS: adenocarcinoma in situ; MIA: minimally invasive adenocarcinoma; IAC: invasive adenocarcinoma; TAM: tumor-associated macrophage; Tregs: regulatory T cells; EGFR: epidermal growth factor receptor

The biological behavior characteristics of pulmonary GGNs are presented in Figure 2.

Pathological types and grading

Pulmonary GGNs primarily represent early-stage lesions of lung adenocarcinoma, with pathological types including atypical adenomatous hyperplasia (AAH), AIS, and MIA. GGNs exhibit significant biological behavior differences depending on their pathological types, which directly influence clinical outcomes. Studies indicate that pGGNs are predominantly associated with AAH and AIS, exhibiting lower invasiveness, whereas MIA and IAC demonstrate stronger invasive capabilities [35,36]. A certain correlation exists between CT imaging features and pathological grading. For instance, nodule diameter, CT attenuation value, density uniformity, and morphological characteristics can reflect different pathological grades and have good predictive value. Specifically, imaging features such as larger nodule diameter (>10.5 mm), greater mean CT attenuation value (>-632 HU), heterogeneous density, irregular shape, coarse margins, lobulation, spiculation, pleural retraction, and vascular dilation and distortion suggest the possibility of invasive lesions [36].

Additionally, the size differences among AAH, AIS, and MIA in pGGNs are not significant, but IACs are typically larger in volume. Clinical studies indicate that pGGNs exhibit an extremely low five-year recurrence rate and relatively favorable prognosis, whereas part-solid nodules (PSNs) and heterogeneous GGNs have a significantly increased risk of recurrence [37]. Therefore, pathological grading not only reflects the biological behavior of nodules but also guides clinical intervention. pGGNs can often be monitored with follow-up, while nodules with solid components may require surgical resection. In summary, detailed grading of pulmonary GGNs based on imaging and pathological characteristics helps accurately assess their malignant potential and guide appropriate clinical management.

Molecular markers and genetic features

The development and progression of pulmonary GGNs are closely associated with various molecular markers. Genetic alterations such as epidermal growth factor receptor (EGFR) mutations, ALK rearrangements, and HER2 mutations are frequently observed in GGNs and are linked to their pathological types and imaging features [38,39]. For example, HER2 mutations are relatively common in subsolid nodules (SSNs), including GGNs, and are more frequently found in smaller tumors and younger patients, suggesting their important role in early-stage lesions [38]. Additionally, EGFR mutations are closely related to the malignant transformation and biological behavior of GGNs, offering potential value for targeted therapy.

miRNAs derived from extracellular vesicles in plasma have also been identified as molecular markers for pulmonary GGNs. Studies using small RNA sequencing have developed miRNA-based support vector machine models that can accurately distinguish malignant GGNs from benign nodules. miRNAs such as miR-500a-3p, miR-501-3p, and miR-502-3p show upregulated expression in tumor tissues compared to normal tissue and are associated with patient survival rates [40]. These molecular markers reveal the molecular diversity of pulmonary GGNs and provide a basis for risk stratification and individualized treatment.

Molecular testing enables precise typing and risk assessment of GGNs, guiding the selection of appropriate targeted therapies for patients with different molecular characteristics and improving treatment efficacy. Furthermore, integrating molecular features with imaging findings can further optimize diagnosis and prognosis prediction. In summary, the molecular markers and genetic characteristics of pulmonary GGNs not only elucidate tumor biology mechanisms but also offer new tools and insights for clinical intervention.

TME and immune status

The biological behavior of pulmonary GGNs is influenced not only by tumor cells themselves but also by immune cell infiltration and inflammatory responses within the TME. Studies indicate that changes in the composition and function of immune cells in GGNs significantly impact malignant transformation [39]. For example, early-stage pGGNs exhibit minimal immune cell infiltration, maintaining a state of strong immune surveillance with weak immune evasion, which may explain their slow growth and favorable prognosis. As solid components increase in GGNs, an immunosuppressive microenvironment gradually develops, with elevated proportions of immunosuppressive cells such as tumor-associated macrophages (TAMs) and regulatory T cells (Tregs), promoting tumor immune evasion and rapid growth [39].

Additionally, inflammatory mediators and cytokines in the TME, such as IL-6 and IL-10 with altered expression levels, also influence the biological behavior of pulmonary GGNs. Research on immune-related markers provides potential targets for immunotherapy, and modulating the tumor immune microenvironment could offer a new strategy for treating pulmonary GGNs. The dynamic changes in the immune microenvironment reflect the complex interactions between the tumor and the host immune system, with differences in malignant potential and treatment response among GGNs under varying immune statuses.

In summary, the TME and immune status are important regulatory factors in the biological behavior of pulmonary GGNs. Studying the GGN immune microenvironment deepens our understanding of malignant transformation mechanisms, helps discover new immunotherapy targets, and advances the development of individualized treatment. In the future, integrating immunological indicators into assessments will enhance the diagnosis and treatment of pulmonary GGNs.

Analysis of the correlation between imaging features and biological behavior

**Figure 3:**
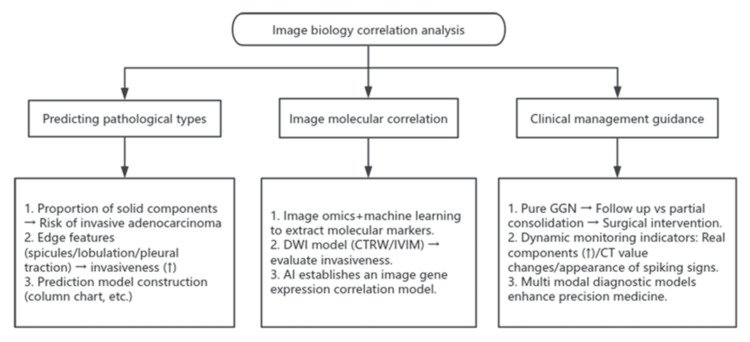
Image Biology Correlation Analysis Flowchart showing the relationship between pulmonary ground-glass nodules (GGNs) and biology, created by Weikuan Xue in Rui'an (2025) DWI: diffusion-weighted imaging; CTRW: continuous-time random walk; IVIM: intravoxel incoherent motion; AI: artificial intelligence

An analysis of the correlation between imaging features and biological behavior is presented in Figure 3.

The value of imaging indicators in predicting pathological types

The imaging features of GGNs are closely related to their pathological types, with imaging findings providing preliminary insights into the nodules' biological behavior. pGGNs typically correspond to non-invasive lesions like AIS or MIA, whereas solid components within nodules suggest potential IAC. Multiple studies indicate that nodule size and margin characteristics are key imaging markers strongly linked to pathological grading. Research shows that larger nodules with higher solid component ratios correlate with increased risk of IAC [41,42]. Furthermore, margin features such as spiculation, lobulation, and pleural indentation show significant associations with tumor invasiveness [43,44]. These imaging characteristics serve as valuable non-invasive diagnostic tools, aiding clinicians in clinical decision-making. For instance, combining quantitative parameters like maximum diameter, solid component ratio, and CT values enables the development of predictive models or nomograms to assess GGN invasiveness, supporting treatment strategies [43,45]. In summary, precise imaging analysis of GGNs is crucial for distinguishing non-invasive from invasive lesions early, advancing imaging's role in lung cancer stratification.

Association between imaging changes and molecular biological features

Dynamic imaging changes in GGNs directly reflect their molecular biological characteristics. Increasing solid components often accompany elevated tumor mutation frequencies, particularly EGFR gene mutations. Studies demonstrate that growth in nodule size and solid components correlates strongly with EGFR mutations, with EGFR-mutated patients exhibiting more complex imaging findings like higher rates of vacuole or honeycomb signs in lung adenocarcinoma [46]. Advanced imaging techniques-including multiphase CT, contrast-enhanced CT, and MRI-can reveal tumor metabolism and proliferation rates, hinting at molecular alterations. For example, diffusion-weighted imaging (DWI) models like continuous-time random walk (CTRW) and intravoxel incoherent motion (IVIM) excel in evaluating tumor invasiveness and histological grading by mirroring the tumor's microenvironment and metabolism [47]. Radiomics and machine learning can identify imaging-based biomarkers tied to molecular biology, enhancing predictive accuracy and reliability [48,49]. AI further establishes correlations between imaging features and gene/protein expression, enabling non-invasive diagnostic precision [50]. Collectively, imaging changes not only capture structural tumor changes but also uncover molecular signatures, providing critical insights for personalized therapy.

Significance of imaging features in guiding clinical management

Imaging-based assessment of malignancy risk in GGNs is essential for developing tailored follow-up and treatment plans. Accurate differentiation between pGGNs and mixed-solid nodules allows optimal monitoring intervals and surgical timing, preventing overtreatment or delays [42,51]. Combining imaging surveillance with biomarker detection (e.g., EGFR/KRAS mutations) refines surgical approaches and treatment strategies, improving patient outcomes [46]. During follow-up, increases in solid components, CT value fluctuations, or emerging spiculation signal heightened invasiveness, warranting timely intervention [43,44]. Multimodal diagnostic models integrating AI and radiomics boost sensitivity and specificity, supporting precision oncology [52]. Radiologic measurements could not precisely predict tumor invasion and prognosis [53]. Multimodal models integrating imaging, intraoperative frozen section pathology information, and clinical indicators can effectively predict the invasiveness of early adenocarcinoma of the lung, guiding surgical strategies during surgery and promoting the development of precision oncology practices [54]. Thus, leveraging imaging features to guide clinical management represents a pivotal direction in personalized lung cancer care.

## Conclusions

GGNs are important imaging manifestations of early lung cancer, and their imaging characteristics are closely related to pathological types and molecular characteristics. Combining imaging and biological information can significantly enhance diagnostic sensitivity and specificity, optimizing clinical management strategies. Current research shows consensus on the correlation between GGN imaging and pathology, but there are still differences in the application of molecular markers and multimodal integration, mainly due to the diversity of sample sizes, technologies, and research designs. Future studies need to be conducted through multicenter prospective research, integrating advanced imaging technology and molecular detection, to clarify the biological behavior patterns of different types of GGN, laying the foundation for precise diagnosis and treatment.

Dynamic imaging monitoring combined with molecular marker detection is an important practice in precision medicine, which can reflect nodule changes in real time and reveal their biological mechanisms, effectively supporting clinical decisions. Promoting the deep integration of multimodal imaging and molecular biology, along with the introduction of AI and big data analysis, will facilitate the development of early lung cancer screening and personalized treatment, ultimately improving patient survival rates and quality of life.
